# Genetic Landscape of Multistep Hepatocarcinogenesis

**DOI:** 10.3390/cancers14030568

**Published:** 2022-01-23

**Authors:** Haruhiko Takeda, Atsushi Takai, Yuji Eso, Ken Takahashi, Hiroyuki Marusawa, Hiroshi Seno

**Affiliations:** 1 Department of Gastroenterology and Hepatology, Graduate School of Medicine, Kyoto University, Kyoto 606-8507, Japan; htakeda@kuhp.kyoto-u.ac.jp (H.T.); yujieso@kuhp.kyoto-u.ac.jp (Y.E.); takaken@kuhp.kyoto-u.ac.jp (K.T.); seno@kuhp.kyoto-u.ac.jp (H.S.); 2 Department of Gastroenterology and Hepatology, Osaka Red Cross Hospital, Osaka 543-8555, Japan; maru@kuhp.kyoto-u.ac.jp

**Keywords:** hepatocellular carcinoma, genetic analysis, intratumor heterogenerity, whole-genome sequencing, molecular targeted therapy, liver cirrhosis, early HCC

## Abstract

**Simple Summary:**

Hepatocellular carcinoma (HCC) is a major cause of cancer-related death worldwide. As the complex genetic landscape of HCC is considered to compromise the antitumor efficacy of targeted therapy, genetic landscape of hepatocarcinogenesis is necessary to be understood in detail. Here, we summarize the landscape of the liver cancer genome and its intratumor heterogeneity. Recent insight on early genetic alterations in hepatocarcinogenesis, especially those in early HCC and noncancerous liver tissues are also introduced. Importantly recent studies demonstrated that noncancerous liver tissues already possess a variety of somatic mutations. Finally, we summarize recent findings on the multistep accumulation of genetic aberrations throughout liver cancer progression. These genetic aberrations have been used for subtyping of liver cancers, which can be applied for clinical practice in the future.

**Abstract:**

Hepatocellular carcinoma (HCC) is a major cause of cancer-related death worldwide. Although several targeted therapy agents are available for advanced HCC, their antitumor efficacy remains limited. As the complex genetic landscape of HCC would compromise the antitumor efficacy of targeted therapy, a deeper understanding of the genetic landscape of hepatocarcinogenesis is necessary. Recent comprehensive genetic analyses have revealed the driver genes of HCC, which accumulate during the multistage process of hepatocarcinogenesis, facilitating HCC genetic heterogeneity. In addition, as early genetic changes may represent key therapeutic targets, the genetic landscapes of early HCC and precancerous liver tissues have been characterized in recent years, in parallel with the advancement of next-generation sequencing analysis. In this review article, we first summarize the landscape of the liver cancer genome and its intratumor heterogeneity. We then introduce recent insight on early genetic alterations in hepatocarcinogenesis, especially those in early HCC and noncancerous liver tissues. Finally, we summarize the multistep accumulation of genetic aberrations throughout cancer progression and discuss the future perspective towards the clinical application of this genetic information.

## 1. Introduction

Hepatocellular carcinoma (HCC) is one of the most lethal malignancies worldwide [1,2,3]. HCC therapy, including surgical resection, liver transplantation, transcatheter arterial chemoembolization (TACE), percutaneous radiofrequency ablation, percutaneous ethanol injection, and molecular targeted therapy (MTT), is selected based on tumor status and liver function [1,4,5,6]. Whereas the curative treatment strategies such as hepatectomy and liver transplantation have resulted in favorable outcomes in terms of overall or recurrence-free survival times, the survival rate of unresectable HCCs is quite poor, as shown in Table 1 [7,8,9,10,11,12,13,14,15,16,17,18]. Approximately 10 years after the introduction of sorafenib, the first approved targeted therapy agent for HCC [17], the non-inferiority of lenvatinib with regard to overall survival (OS) was demonstrated in a phase III trial, resulting in the latter’s inclusion in clinical practice as another choice of first-line systemic therapy [18,19,20]. Multi-kinase inhibitors regorafenib and cabozantinib, as well as the VEGFR2 inhibitor ramucirumab, have been approved as second-line MTT following sorafenib treatment [8,21].

Immune checkpoint inhibition has also been introduced, with PD-1 inhibitor nivolumab being the first checkpoint inhibitor approved by the FDA for the treatment of HCC in patients who have been previously treated with sorafenib. Of note, another PD-1 inhibitor, pembrolizumab, was approved for any type of cancer with high microsatellite instability (MSI-high), HCC included [22]. In addition to regimens utilizing immune checkpoint inhibitors (ICIs), recently developed regimens utilize a combination of ICI and other molecular targeting agents. The IMbrave150 trial demonstrated that dual therapy using atezolizumab, a PD-L1 antibody, with bevacizumab, which is an antibody directed against VEGF, significantly improved OS, progression-free survival (PFS), and the objective response rate (ORR) compared to monotherapy using sorafenib as the first-line chemotherapeutic agent for patients with advanced HCC [9].

Although the treatment options for patients with advanced HCC have been increasing [23], the antitumor efficacy of these approved agents remains limited. In the IMbrave150 trial that showed among the most favorable results for HCC to date, the ORR assessed using RECIST1.1 after the administration of atezolizumab with bevacizumab was 27.3% [9]. Among alternate choices for patients with advanced HCC, the ORR after administration of sorafenib was less than 5% in the SHARP trial [17] and was limited in clinical practice [24,25,26,27,28], whereas the ORR for lenvatinib was 18.8% in the REFLECT trial [18]. Although recent advances in systemic therapies for HCC are remarkable [29], there have been no established treatment strategies effective for every patient with advanced HCC to date [30,31].

While considering the development of a novel therapeutic strategy for liver cancer, it is essential to understand the landscape of accumulating genetic aberrations in liver cancer cells [32]. Utilizing recently developed next-generation sequencing (NGS) approaches, international projects have carried out comprehensive genetic analyses of liver cancer, identifying several driver genes associated with liver carcinogenesis [33,34,35,36,37,38]. Recent molecular biology studies have identified molecular pathways associated with liver cancer [3,39,40]. Additionally, mechanisms underlying the accumulation of several cancer-related genetic aberrations in tumor cells during multistep liver carcinogenesis have been investigated. In this review article, we summarize the recent investigations on the genetic alterations that accumulate during the multistep process of hepatocarcinogenesis, ranging from precancerous liver tissues and early HCC to advanced HCC (Table 2).

## 2. Genetic Landscape of HCC

### 2.1. Comprehensive Genetic Analysis of HCC

As evidenced by the International Cancer Genome Consortium (ICGC) and The Cancer Genome Atlas (TCGA) project [48,49], vast amounts of information on genetic aberrations across various types of tumor tissues have been collected worldwide. In addition, whole-exome and genome analyses of liver cancer, including HCC, have been recently carried out in different countries, elucidating the greater picture of genetic aberrations accumulated in transformed liver tissues [50].

**Figure 1:**
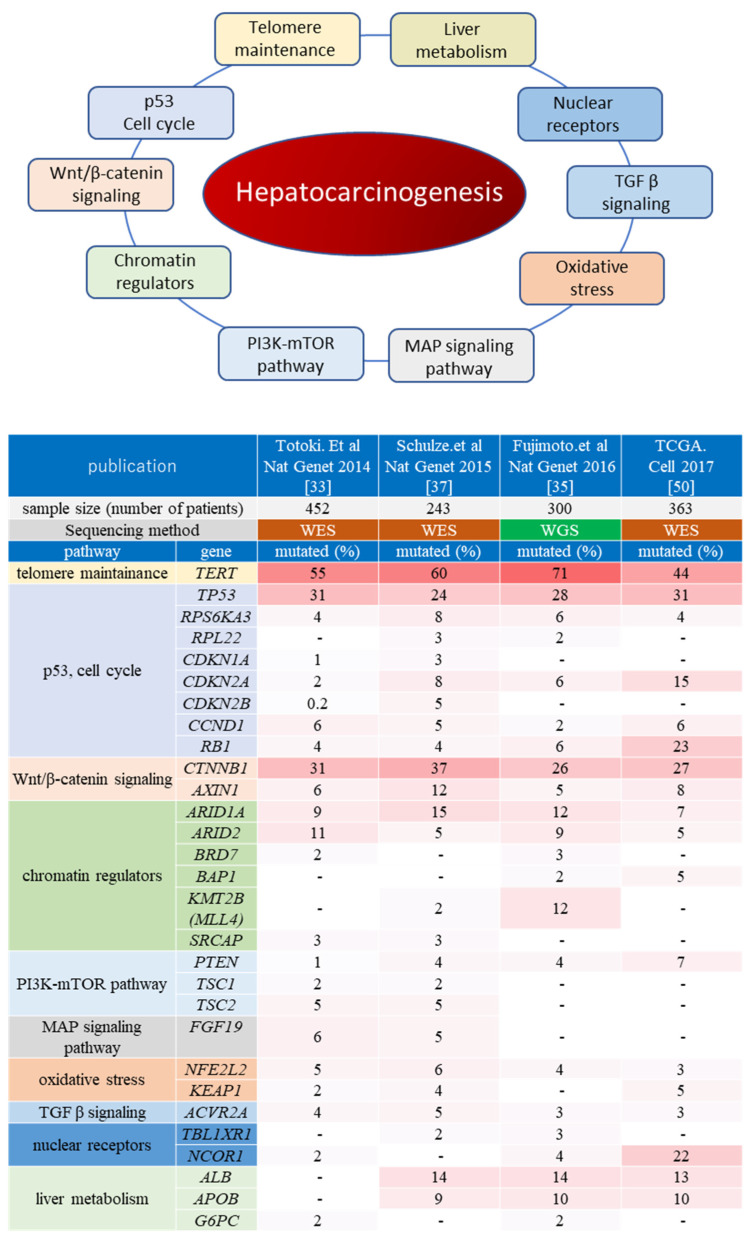
Oncogenic pathways associated with hepatocarcinogenesis. The upper panel shows the major oncogenic pathways elucidated via comprehensive genome analysis projects. The lower heatmap indicates the genes which have been reported as putative liver cancer driver genes in at least two publications of ICGC/TCGA projects. The top row shows each publication, and the second row shows the number of liver cancer samples analyzed. The major pathways and representative cancer-related genes for each pathway are listed on the left. The mutation frequency of each gene (percentage of the cases with mutated genes among all cases analyzed in each cohort) is shown as a heatmap. WES: whole-exome sequencing; WGS: whole-genome sequencing.

Large-scale whole-exome sequencing (WES) of the liver cancer genome has been conducted mostly in the United States (TCGA) [50] as well as in Japan, China, and France (ICGC) [33,36,37,38,51], while whole-genome sequencing (WGS) has been performed mainly in Japan. WGS analyses revealed over 9000 point mutations per human liver cancer sample, with somatic mutations detected in approximately 40–80 protein-coding genes per specimen [34,35,52]. Previous genetic analyses have detected a number of candidate HCC driver genes (Figure 1). 

In addition to coding mutations, noncoding mutations have also been detected, and some HCC candidate driver mutations have been identified among them, such as the promoter sequence of the *TERT* gene, as well as long intergenic noncoding RNAs [35,53]. Various aberrations can be observed in the *TERT* promoter region, including hotspot point mutations, structural alterations, as well as hepatitis B virus HBV genome integration. Importantly, *TERT*-associated genetic alterations are found in over 70% of HCC tissues, making them the most frequent aberrations associated with HCC [35].

WES/WGS analysis confirmed *TP53* and *CTNNB1* as the most frequently mutated coding genes in HCC, with chromatin modulators *ARID1A* and *ARID2* also recurrently mutated [33,37]. Apart from frequently observed mutations in these driver genes, many HCC-related mutated genes have been identified in cancer patients, with a frequency of less than 5% among cases described by the so-called long-tail distribution of driver genes, which facilitates the genetic heterogeneity of HCC [33].

Mutational signature analyses following whole-exome/genome sequencing [54] have revealed that the most frequent patterns of single nucleotide changes in HCC are C>T/G>A as well as T>C/A>G. Some liver cancer-specific mutational signatures, such as signatures 12 and 16 in the Catalogue Of Somatic Mutations In Cancer (COSMIC) Mutational Signature, have been identified, although the etiologies of these signatures remain unknown [33,55]. Of note, the whole-exome analysis revealed differences in mutational signatures according to geographical origin among European, Asian American, and Japanese people. The correlation between specific driver gene mutations and genome-wide mutational signatures has also been demonstrated [33]. Thus, comprehensive genetic analyses in different patient populations have elucidated the landscape of genetic aberrations accumulated in liver cancer cells.

### 2.2. Molecular Pathways Associated with Hepatocarcinogenesis

Hepatocarcinogenesis-associated molecular pathways have been summarized in detail previously [56]. As described in the above section, *TERT*-associated genetic alterations are the most frequently detected in HCC. Nevertheless, various other molecular pathways have been demonstrated to contribute to hepatocarcinogenesis. For example, Schulze et al. summarized HCC-related genetic alterations into eleven categories, including telomere maintenance, Wnt/β-catenin signaling, p53/cell cycle, oxidative stress, epigenetic regulation, PI3K-Akt-mTOR, MAPK, and hepatic differentiation [37]. Among them, genes involved in Wnt/β-catenin and p53/cell cycle signaling are frequently aberrated in HCC. These findings suggested that HCC is not caused by one particular driver mutation but involves several oncogenic pathways, making tumors highly heterogeneous [56]. Herein, we summarize the main pathways and processes involved in hepatocarcinogenesis (Figure 1).

#### 2.2.1. Telomere Maintenance

Telomerase reactivation is a major event in malignant transformation. As in various other malignancies, telomerase expression is known to be increased in most HCC tumors [35,53,57]. The causes of telomerase reactivation include hotspot mutations in the *TERT* promoter sequence (54–60%), gene amplification (5–6%), structural variation (4–5%), and HBV integration in the promoter or gene body (10–15%). In general, these aberrations are mutually exclusive [35].

Another mechanism of telomerase reactivation is through epigenetic changes. Hypermethylation of specific regions has been confirmed as associated with *TERT* mRNA expression in breast, colorectal, and other cancers [58,59,60]. We previously demonstrated the aberrant methylation status of the *TERT* promoter in HCC tissue, along with elevated *TERT* mRNA expression levels [46].

#### 2.2.2. Wnt/β-Catenin Pathway

The Wnt/β-catenin pathway is one of the most frequently activated oncogenic pathways in HCC. It is usually upregulated by activating *CTNNB1* mutations or inactivating mutations of the *AXIN1, NCOR1,* and *APC* genes [32,61,62]. These mutations were detected in a mutually exclusive manner. Interestingly, previous WES studies revealed that the CTNNB1 mutation tended to co-occur with *TERT* mutations [33]. Although *AXIN1* has been generally described as a well-known negative regulator of the Wnt/β-catenin pathway, it is notable that Abitbol et al. recently reported that *AXIN1*-mutated HCCs occur independent of the Wnt/β-catenin pathway and involve Notch and YAP pathways [63].

*CTNNB1* mutations are sometimes considered when molecular subclasses of HCC are classified. Importantly, *CTNNB1* is the only gene with a mutation that defines a specific subclass of HCCs (the perivenous-type HCC subclass [64]), with a particular metabolic zonation phenotype or G5/G6 subtypes in Boyault’s classification [65]. Tumors carrying *CTNNB1* exon three mutations are generally moderately differentiated and of low-to-intermediate aggressiveness [66].

#### 2.2.3. p53/Cell Cycle

p53/cell cycle signaling is altered in at least half of HCC patients with *TP53* mutations. In general, tumor suppressor genes are more frequently mutated in cancer. The retinoblastoma (RB) pathway, which controls progression from the G1 to the S phase of the cell cycle, is inactivated in HCC mainly as a result of homozygous *CDKN2A* deletions or *RB1* mutations. Among the genes involved in this pathway, recurrent HBV insertions have been reported in *CCNE1* (coding cyclin E1). In addition, the focal amplification of *CCND1/FGF19*, which is downstream of this oncogenic pathway, has been reported in over 10% of HCC tissues. A recent study demonstrated that some HCC subgroups exhibit cyclin activation through various mechanisms, including HBV and adeno-associated virus type 2 (AAV2) insertions and enhancer hijacking and recurrent *CCNA2* fusions, defining a homogenous entity of aggressive HCC [67].

Importantly, TP53 mutations have been demonstrated to be related to the aggressiveness, some phenotypes, and etiologies of HCC. For example, *TP53* mutations are associated with aggressive HCCs of the STEM (Désert’s classification) [64] S2 (Hoshida’s classification) [68] or G3 (Boyault classification) subclasses [65]. These tumors tend to be associated with HBV infection and genomic instability [69]. Moreover, there is an etiological link between *TP53* mutations and aflatoxin DNA adducts in intertropical regions of the World [70].

#### 2.2.4. Chromatin Remodeling Factors

Alterations in chromatin remodeling factors, such as *ARID1A, ARID1B, ARID2*, and *BRD8*, are also considerably associated with hepatocarcinogenesis [33,37]. In particular, mutations of *ARID1A* and *ARID2*, which encode key players in SWI/SNF chromatin remodeling complexes, are frequently detected in liver cancer [71]. These complexes modify the chromatin structure and nucleosome position, thus indirectly regulating the determination of cell fate. Recurrent somatic alterations in genes encoding histone methylation writer family proteins, mainly MLL1-4, are also observed in HCC. Notably, the *MLL4* gene is known to be the second most frequent region of HBV integration in HCC [72,73].

#### 2.2.5. PI3K/Akt/mTOR and RAS/RAF/MAPK Pathways

Activating mutations of *PIK3CA* and inactivating mutations of *TSC1* as well as *TSC2* lead to the activation of Akt/mTOR signaling in a subset of HCC tumors. In addition, homozygous deletion of *PTEN*, which encodes an inhibitor of the PI3K kinase, has been identified in 1–3% of HCC cases [33,35,37].

Activating mutations in genes belonging to the RAS family are rarely observed in HCC (<2%), and inactivating mutations of *RP6SKA3*, encoding RAS inhibitor RSK2, were identified in 2–9% of liver cancer cases. The inactivation of RSK2 releases associated negative feedback, inducing constitutive activation of the pathway. In approximately 5–10% of HCC cases, the PI3K/Akt/mTOR and RAS/RAF/MAPK pathways are upregulated through amplification of the *FGF19/CCND1* locus [33,35,37].

#### 2.2.6. Other Oncogenic Pathways in Hepatocarcinogenesis

Genes associated with oxidative stress are altered, such as activating mutations in *NFE2L2* (encoding NRF2) or inactivating *KEAP1* mutations in 5–15% of liver cancer cases [33,35,37]. Further, genes associated with the IL-6/JAK-STAT pathway, TGF-β signaling, and liver differentiation are also altered in some cases of liver cancer [33,35,37]. Interestingly, WGS revealed that mutational clusters accumulated in *ALB* (encoding albumin) or *APOB* (encoding apolipoprotein B), although these were not directly associated with cancer development. Most *ALB* mutations are short indels in the gene body, whose significance with regard to hepatocarcinogenesis has not been elucidated [35].

Some driver genes discovered by WGS projects were characterized based on their focal amplifications, including *VEGF* (coding vascular endothelial growth factor), *MYC*, and *MET*, leading to overexpression of their mRNA, which results in the activation of various oncogenic signaling pathways [74]. As one of the most typical steps during multistep hepatocarcinogenesis, the acquisition of a hypervascular feature comprising genes associated with angiogenesis, is considered important when examining its pathogenesis and molecular targets. Especially, *VEGFA* amplification has been discussed as a biomarker to predict the treatment effect of some molecular targeting therapies [24,75]. Notably, analyzing the WGS data has discovered some noncoding drivers in HCC. Fujimoto et al. reported two long intergenic noncoding RNAs (lincRNAs), *NEAT1* and *MALAT1*, as significantly mutated drivers in HCC tissues [35], whereas a recent pan-cancer WGS analysis revealed that U1 small nuclear RNAs (snRNAs), the noncoding component of the spliceosome, are also mutated, suggesting that driver genes or locus can exist over a wider range of genomic regions [76].

## 3. Intratumor Genetic Heterogeneity

### 3.1. Intratumoral Heterogeneity Revealed by NGS

To date, phylogenetic analyses utilizing multiregional sequencing methodology have been employed for the analysis of various cancer types, and the modes of cancer evolution have been revealed to differ among various cancers [77]. The modes of evolution include some classical evolutional models, including a linear evolution model with sequentially acquired driver mutations (late diversification) [78] or branched evolution with many standing lineages (early diversification) [79,80,81]. For example, Gerlinger et al. explored the intratumor heterogeneity of metastatic renal cell carcinoma through multiregional sequencing [79]. They demonstrated the branched evolution of cancer cells, where some mutations acquired by the parental cell at the early stage of tumorigenesis are inherited by the majority of tumor cells and are called “ubiquitous” mutations, while mutations acquired by each subclone in the comparatively late phase of tumorigenesis are called “shared” or “private” mutations in a phylogenetic tree. Ubiquitous mutations are also described as trunk mutations, whereas private or shared mutations are also described as branch mutations [82]. These evolutionary processes lead to the formation of subclones in each tumor, generating intratumor clonal diversity, or so-called intratumor heterogeneity [79,81]. Similarly, intratumor and intertumor genetic heterogeneity have been identified in colorectal cancer, glioblastoma, and many other malignancies [80,83,84,85,86,87].

Intratumor heterogeneity might explain the difficulties encountered in the validation of oncology biomarkers owing to sampling bias. For example, in the multiregional sequencing analysis of RCC by Gerlinger et al., 63–69% of all somatic mutations found based on multiregion sequencing were heterogeneous and not detectable in every sequenced region [79]. Multiregional WES analysis of HCC by Lin et al. demonstrated a median of 38.9% (range: 5.5–91.7%) of heterogeneous variants, and the genetic heterogeneity could also be validated at the protein level by immunohistochemistry [83].

It should be noted that when branch mutations are targeted by certain drugs, the therapeutic effect may be limited to the subpopulations harboring the branch mutation. Importantly, if trunk mutations shared by most of the tumor cells are identified, the effect of therapy targeting these mutations is expected to be favorable. Gerlinger et al. suggested that reconstructing tumor clonal architectures and the identification of common mutations located in the trunk of the phylogenetic tree might contribute to more robust biomarkers and therapeutic approaches [79]. In this context, it is important to understand the hierarchy of driver mutations that facilitate tumor heterogeneity through cancer cell evolution.

### 3.2. Intratumoral Heterogeneity of HCC

Intratumor heterogeneity has been also explored in liver cancer including HCC [46,80,82,88,89,90,91,92]. Lin et al. performed multiregional WES on large tumor tissues from 10 HCC patients and constructed a phylogenetic tree of each tumor, showing that identical liver nodules harbored a variety of subclones [82]. Moreover, Zhai et al. reported a multiregional WGS or WES study on nine HCC patients, whereas Xue et al. conducted a phylogenetic analysis of 53 samples from 10 patients with HCCs. They described a variety of evolutionary trees with different degrees of intratumoral heterogeneity, most of which exhibited the branched evolution in HCC [80,88]. Every report demonstrates that the numbers of passenger mutations and some driver mutations are shared by all different regions of a single tumor, suggesting that intratumor heterogeneity in HCC can develop from a single origin cell.

**Figure 2:**
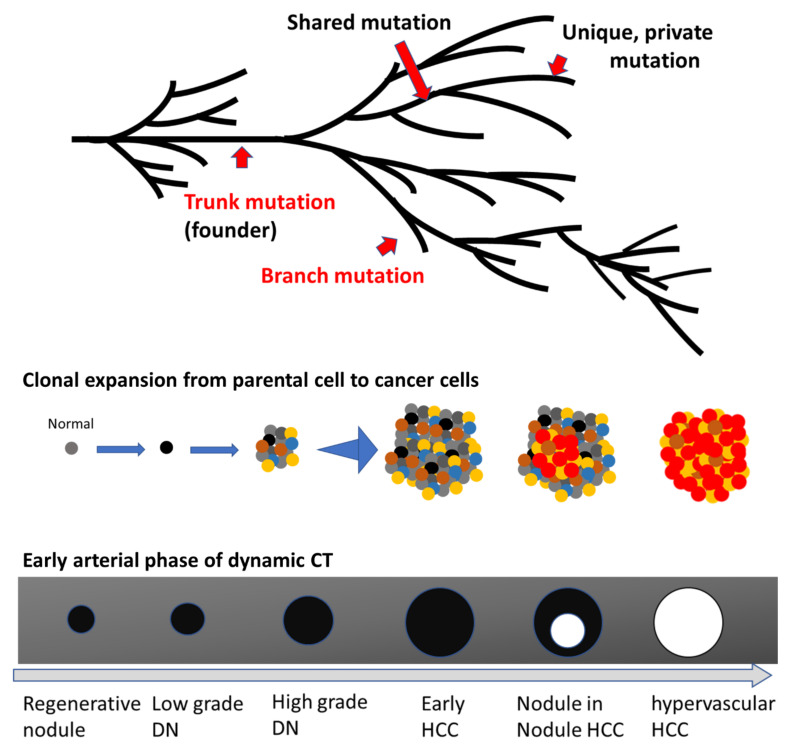
Scheme of the multistep evolution of typical hepatocellular carcinoma (HCC) The upper scheme is a phylogenetic tree showing a scenario of the progression from a normal cell to an advanced HCC. Examples of trunk and branch mutations in addition to shared as well as unique mutations are shown by red arrows. The middle scheme depicts the evolutionary progression from parental cancer cell to dysplastic nodule (DN), early HCC, classical HCC, and, finally, metastatic HCC. Each colored dot indicates a tumor cell with a different mutational profile, and each nodule consists of various tumor cells with distinct genetic alterations, contributing to intratumoral heterogeneity. The red dots indicate progressive tumor cells. This scheme omits nonepithelial cells such as vessels and fibrotic tissues. The lower scheme describes the typical radiological change of liver nodules during multistep hepatocarcinogenesis via nodule-in-nodule HCC, shown in the early arterial phase of contrast-enhanced dynamic computed tomography (CT) imaging. The black areas indicate nodules described as hypovascular areas in the early arterial phase of dynamic CT, whereas white circles mark hypervascular nodules in the early arterial phase of dynamic CT.

Based on the branched evolution model of HCC, a schematic evolutionary tree is shown, along with radiological findings of multistep hepatocarcinogenesis, in Figure 2. In this evolutionary model, a normal parental cell acquires certain trunk mutations associated with the initiation of malignant transformation, followed by the emergence of a single parental cancer cell. Thereafter, this parental cell acquires additional genetic aberrations, including driver and passenger mutations, clonally expanding with the numbers of subclones [69,70,81]. During this multistep cancer cell evolution, many subclones emerge in a single nodule, contributing to intratumor heterogeneity [81].

Regarding driver genes associated with HCC, many mutations were detected heterogeneously in some reports of multiregional sequencing. However, by calculating the cancer cell fraction of all mutations, Lin et al. demonstrated that several driver mutations are fully clonal within some tumor regions. They reported that *TP53* mutations are likely detected as trunk mutations, although less than half of HCC cases had TP53 mutations. *TERT* promoter mutations were almost always detected ubiquitously [80,82]. Other driver genes can be detected as trunk mutations, such as *ARID1A* and *SETD2*, but the mutated trunk mutations vary across cases, which can be considered intertumor genetic heterogeneity [80].

Multiregional analysis of HCC has also been conducted using RNA sequencing, as well as proteome analysis, revealing the complex nature of HCC tumor heterogeneity [80], which might complicate the treatment of advanced HCC via targeted therapy. Furthermore, recent progress in single-cell sequencing technologies has enabled an understanding of the heterogeneity of microenvironments of HCC tissues. These analyses have demonstrated the ecosystem of HCC, including not only cancer cells but also infiltrating immune cells. As the novel therapeutic strategies for HCC include immunocheckpoint inhibitors (ICIs), exploring the single-cell level heterogeneity, including immune cells, should have significance for translational research, such as the prediction of ICI treatment efficacy.

## 4. Genetic Alterations in Early Hepatocarcinogenesis

### 4.1. Genetic Landscape of Early HCC

The heterogeneity described in the previous section is based on the genetic landscape of classical surgically resected HCCs, arising as the result of multistep carcinogenesis. Considering the importance of early HCC detection, it is essential to understand the genetic landscape during the early stages of hepatocarcinogenesis.

Liver cancer usually develops from liver cirrhosis, and after a dysplastic premalignant nodule is initially formed, it becomes early HCC (eHCC), followed by progression into hypervascular classical HCC. Several studies have explored the relatively early stages of multistep hepatocarcinogenesis. In particular, the genetic characteristics of early HCCs were recently reported by Nault et al. [47]. Using Sanger sequencing, they analyzed *TERT* promoter hotspot mutations, observing that approximately 60% of eHCC cases harbored *TERT* mutations, while nearly 65% of classical HCC tumors had *TERT* promoter mutations. This clearly showed that many eHCC have already acquired point mutations in the *TERT* promoter region at this early stage. In contrast, mutations in other HCC-associated driver genes, such as *TP53* and *CTNNB1*, were not detected in the eHCC samples. It should be noted that, due to the low sensitivity of Sanger sequencing, this analysis may have missed somatic mutations with low variant allele frequencies. Thus, it cannot be concluded whether certain cancer cells with driver mutations other than those of the *TERT* promoter mutations existed in the analyzed eHCC tissues.

### 4.2. Genetic Landscape of Precancerous Liver Tissues

To elucidate genetic aberrations developing in the earliest step of hepatocarcinogenesis, we previously conducted a genetic analysis of nontumor liver cirrhosis tissues, from which liver cancer cells originate [43]. To this end, we focused on the genetic alterations accumulated in the regenerative nodules (RNs) of cirrhotic livers. While the so-called bulk sampling from normal tissue is likely to fail in the detection of somatic mutations at the single-cell level, RNs have been previously demonstrated to possess clonal structures [93,94,95,96], which justified the genetic analysis of these structures. Based on WES, the somatic mutation rate of RNs was approximately one-tenth that of HCC, and the most enriched mutational signature was COSMIC signature 1 [97], mimicking that of HCC. The majority of the somatic mutations forming COSMIC signature 1 in the regenerative nodules did not include HCC-associated driver genes but passenger mutations, which is considered reasonable because COSMIC signature 1 is a clock-like or aging-related signature. Targeted deep sequencing of HCC-associated driver genes demonstrated that several damaging nonsynonymous mutations with low allele frequencies were acquired in RNs. One of the genes recurrently mutated in several nodules was major HCC-related chromatin remodeling factor ARID1A. None of the 205 RNs analyzed by targeted deep sequencing harbored *TERT* promoter mutations, and the *TERT* mRNA levels of RNs were as low as those in healthy liver tissue. These results suggested that RNs already accumulate several genetic alterations while their telomerase activity remains suppressed.

Similarly, two large-scaled genetic analyses have been conducted on cirrhotic liver tissues (Table 2). Zhu et al. carried out a genetic analysis of non-tumor cirrhotic liver tissues of METAVIR stage F1 to F4 [42]. Using WES as well as targeted sequencing, they identified *PKD1, KMT2D, PPARGC1B*, and *ARID1A* as significantly mutated genes in cirrhotic liver tissues. The loss of these genes was considered regeneration-promoting and hepatoprotective. This concept resembles the suggestion of a previous report stating that *NOTCH1* mutations frequently detected in normal esophageal tissues exert a tumor-suppressive effect [98].

In addition, Brunner et al. conducted a WGS study on 482 microdissections from five normal and nine cirrhotic liver tissues [41]. 

In this study, multiregional sampling was performed using a single RN. The different regions of this RN shared common somatic mutations, which differed from those detected in neighboring RNs. These observations raised two important points, the first being that a hepatocyte acquiring a specific mutation proliferates in the RN and goes on to form a clonal cell population. Second, clonal expansion does not cross over the fibrotic tissues between RNs, creating the individual mutational landscape of each RN. Although regenerative nodules are not neoplastic tissues, multiregional WGS clearly revealed their clonal structures. Comparison of mutational profiles between regenerative nodules and normal livers revealed that the cirrhotic liver not only has a higher mutational burden but also harbors various structural variants, including ones that have undergone chromothripsis. These results indicated that inflamed nontumor liver tissues, such as the RNs, have already acquired various genetic aberrations prior to malignant transformation, although most of these somatic mutations are considered passenger mutations or mutations contributing to an inflammatory microenvironment adaptation. It should be noted that *TERT* promoter mutations or other *TERT*-associated genetic alterations were not observed in RNs, supporting the previously discussed multiregional genetic analysis of cirrhotic liver tissues. Considering these data, even if hepatocytes acquire somatic mutations under chronic inflammation, they may not evolve into malignant cells. As many researchers have indicated, genetic aberrations associated with the *TERT* gene should be the key event in malignant transformation, with the reactivation of telomerase being one of the first steps in hepatocarcinogenesis.

However, *TERT* promoter hotspot mutations can be detected in 6–19% of dysplastic nodules, whereas 10 other well-known driver gene mutations could not be detected by Sanger sequencing [47]. To date, only a little is known about the genetic landscape of dysplastic nodules, and there have been no whole-genome or whole-exome sequencing data for liver dysplastic nodules to date. Genetic analysis of low-grade and high-grade dysplastic nodules in comparison to regenerative nodules and chronic hepatitis tissues is therefore essential for a deeper understanding of the genetic landscape during early-stage hepatocarcinogenesis.

## 5. Multistep Acquisition of Genetic Aberrations during Hepatocarcinogenesis

### 5.1. The Significance of Comprehensive Analysis Using Clinical and Genetic Data

A multiregional tumor genetic analysis explored intratumoral heterogeneity in HCC [80,82], revealing that most tumor cells share trunk mutations, whereas subclones within the same tumor eventually attain various branch mutations. As these multiregional sequencing analyses were based on random sampling without consideration of the biological features of obtained tissue specimens, it is unclear whether the branch mutations detected in these multiregional sequencing analyses were driver mutations or simply “passenger” nucleotide alterations.

There are apparent differences in biological characteristics between early-stage HCCs, which are detected as hypovascular nodules in the early arterial phase of dynamic CT or EOB-MRI imaging, and classical HCCs, which are characterized by hypervascularity in the early arterial phase and hypovascularity in the portal phase of dynamic CT or EOB-MRI imaging. As the therapeutic strategies for hypovascular early HCC and hypervascular classical HCC differ in clinical practice, it is important to determine which branch mutations drive the progression from hypovascular early HCC to hypervascular classical HCC. To assess this, mutation data from each specimen should be accompanied by radiological, pathological, as well as clinical information.

### 5.2. Whole-Genome Mutational Analysis Using Nodule-in-Nodule HCCs

In order to elucidate the multistep accumulation of genetic aberrations in a single liver tumor, we focused on rare HCC phenotypes with nodule-in-nodule appearance (NIN-HCC), consisting of hypervascular (progressed) HCC surrounded by hypovascular (early) HCC arising from a common origin. Whereas there are various progression patterns for HCCs, such as the confluent multinodular type and infiltrative type for which the progression pattern could be different from the NIN evolutional pattern, NIN-HCC has been a good model to explore multistep carcinogenesis to date. Transcriptomic and genome-wide copy number analyses have been previously carried out in NIN-HCC samples, revealing differences in expression and copy number alterations between early and progressed HCC [44,99]. We conducted multiregional WGS of NIN-HCCs and compared genetic alterations between hypovascular and hypervascular HCC in identical nodules [46]. Based on the genetic landscape, together with pathological and radiological findings, we examined the stepwise evolution of cancer cells from slow-growing hypovascular HCC cells to classical HCC.

Phylogenetic analysis revealed thousands of point mutations, and even several structural variations were identified as trunk mutations at the early stage of hepatocarcinogenesis. Among them, *TERT*-associated aberrations were present as trunk mutations in all cases examined, including point mutations in the promoter region, chromosomal translocations, or HBV integration. Consequently, *TERT* mRNA levels were significantly elevated in almost all cases. Genetic aberrations and *TERT* reactivation are considered key events in the initiation of liver carcinogenesis. In some cases, chromothripsis, which is a large-scale genome rearrangement with a focal accumulation of structural variations such as translocations, duplications, inversions, and deletions, is detected at the trunk level. It is important that such a catastrophic genetic event can occur in the initiation step of early hepatocarcinogenesis.

In contrast, various genes involved in cell growth pathways are aberrantly expressed in hypervascular HCC. Further, these tend to differ between cases. One case harbored LOH of *PTEN* at the early stage of HCC, as well as a second hit of the *PTEN* gene in the form of a nonsense mutation, which was considered to have driven the progress toward hypervascular HCC. In another case, a *CTNNB1* mutation was a hypervascular HCC-specific genetic alteration. Further, genes associated with the Wnt/β-catenin pathway, such as *CTNNB1, AXIN1*, or *HNF4A*, were mutated only in hypervascular aHCC regions with relatively high variant allele frequencies, suggesting that these were associated with tumor progression from hypovascular to hypervascular HCC in the cohort.

These results are of major relevance when considering therapeutic targets in different liver cancer patients. For example, telomerase could be a pivotal candidate drug target for the suppression of hypovascular early HCC development, while proteins associated with Wnt or PIK3CA-mTOR signaling can be targeted to inhibit the progression from hypovascular to hypervascular HCC. Taken together, our findings indicated that the pivotal targets associated with tumor progression differ between cases, highlighting the importance of personalized strategies for the identification of therapeutic targets and the respective MTT for each patient.

### 5.3. Genetic Profiles with Tumor Progression

The above-described multiregional sequencing study focused on the multistep accumulation of genetic aberrations in a single nodule during early disease, that is, within BCLC stages 0 or A. In contrast, Nault et al. analyzed the genetic profiles of hundreds of HCC tissues of BCLC stage 0, A, B, and C [100]. They compared genetic landscapes between advanced-stage HCC and BCLC 0/A tumors. Somatic alterations of *SF3B1, RB1*, and *TP53* were enriched in advanced-stage tumors, whereas *CTNNB1* mutations were more frequently detected in BCLC 0/A HCCs. Notably, the mutation frequencies of other classical genetic drivers, such as the *TERT* promoter, *ARID1A*, and *ARID2*, did not differ between advanced and early BCLC stage HCCs.

There have been several combined transcriptomic–genomic classifications of HCCs, some of which are tightly associated with tumor progression. Désert et al. recently analyzed a 1,133-HCC transcriptomic metadata set along with validation based on a 210-HCC RNA-sequencing set, classifying all HCCs into four categories, namely periportal (PP), perivenous (PV), extracellular matrix (ECM), and STEM (displaying stem cell features) subclasses [64]. From the molecular profiling, they demonstrated the existence of increasingly aggressive tumor phenotypes from PP through PV, ECM, and STEM subtypes. *CTNNB1* mutations are frequently detected in the PV subtype, which is a well-differentiated HCC subclass carrying mutant *CTNNB1* and expressing β-catenin target genes. *CTNNB1* mutations are also enriched in the G5/G6 type in Boyault’s classification and Hoshida’s S3 subtype [68], either of which fit within the non-proliferative HCC class [32]. However, *TP53* mutations are more frequent in poorly differentiated tumors, such as the STEM subtype in Désert’s classification [64], the S2 in Hoshida’s system [68], and G3 in Boyault’s classification [65]. Interestingly, BCLC classification, from stages A to C, also follows transcriptomics classification from the periportal-type to STEM-type of Désert’s classification [64]. In this manner, recent molecular subtype classifications are closely associated with clinical phenotypes associated with the multistep progression of HCC.

### 5.4. Multi-Omics Analyses of Multistep Hepatocarcinogenesis

Different levels of omics data on cancer development are intricately associated with each other. For example, the gene expression profile is influenced not only by mutations but also by epigenetic modifications as well as other mechanisms, and some reports have demonstrated that proteomic and transcriptomic HCC data often do not correspond to mutational data. In light of these observations, omics approaches other than DNA sequencing are essential in order to understand the molecular basis of multistep hepatocarcinogenesis. In this context, Midorikawa et al. comprehensively compared the whole-exome mutational landscape as well as transcriptomic and epigenetic profiles between early and advanced (overt) HCC. They classified 191 HCC cases using RNA-seq and BeadArray data observing that Wnt target genes, as well as target genes downstream of the p53/RB pathway (cell cycle-related genes), were rarely upregulated in early HCC, despite the frequent mutations of *CTNNB1* and *TP53* at that stage. These findings highlight that mutational data alone are not sufficient to explain changes in pathway activity. Downregulation of the *CDH1* gene and the chromosomal deletion of the 4q and 16q arms were associated with the transcriptional activation of downstream targets of the Wnt and p53/RB pathways, respectively, during HCC progression. Multi-omics analysis using early and advanced tumor regions of NIN-HCC tissues was also conducted, and the multistep genetic and transcriptomic alterations during HCC progression were validated. In contrast, interestingly, the global methylation status was found to be maintained throughout HCC progression.

Based on the methylation status and mutational/transcriptional profiles, patients were categorized into four groups (G1–G4): the G1 (normal-like) grouping included patients with certain HCC-related gene mutations such as in *TP53* and *CTNNB1* during the progression phase, whereas the G2 (global hypomethylation) group showed mutations in *TP53* at early stages and subsequent 4q or 16q loss during the progression phase. Patients in the G3 group (stem-like methylation) were characterized by mutations in *TP53*, EZH2 upregulation and showed poor survival. The G4 group (CpG island methylation) included patients with *TP53* and *CTNNB1* mutations during the progression phase. In each case, genetic and transcriptional characteristics in the progression phase included the upregulation of genes related to the cell cycle, Wnt target gene activation, and cellular growth. Further, the immortalization of cells by *TERT* upregulation was shown to be a common aberration at the initiation step in all subgroups evaluated [78].

Taken together, a two-step classification of genetic aberrations in multistep hepatocarcinogenesis can be performed; the first step includes *TERT*-aberration associated cell immortalization, and the second step involves the abnormal upregulation of genes associated with the cell cycle and cell growth pathways caused by genetic and transcriptional aberrations (Table 1).

## 6. Conclusions and Perspectives

In the current work, we summarized the genetic landscape throughout multistage hepatocarcinogenesis, including precancerous tissue, early HCC, and advanced HCC. In the early stages of tumorigenesis, hepatocytes acquire *TERT*-associated genetic aberrations and are immortalized. Subsequently, various genes associated with cell cycle regulation, chromatin remodeling, and growth-associated signaling pathways are dysregulated in the immortalized parental cell. The accumulation of these genetic and expression changes drives the progression of early to advanced HCC, complicating the tumor’s genetic profile. Intratumoral HCC heterogeneity is thus established, compromising targeted molecular therapy. In order to understand the molecular mechanisms underlying multistep hepatocarcinogenesis, the application of multiregional and multi-omics strategies is crucial, and thus further investigations utilizing novel sequencing technologies are warranted.

Importantly, genome-based precision medicine has already been introduced into the clinic, supporting the decision-making process of anti-cancer drug selection. Nevertheless, even though a number of agents that target the products of genetic aberrations have been developed, only a few patient subgroups benefit from those in the clinic. Recent whole-genome multi-omics analyses data have clearly answered for the reason why the current genome-based medicine has comparatively low efficacy. First, sequencing data obtained via targeted sequencing is only a small part of the whole-genome mutational profile, potentially overlooking various other genetic aberrations associated with tumor initiation and progression. Second, mutations detected by targeted sequencing include certain trunk mutations together with many branch mutations, some of which are not crucial for tumor progression. Further, bulk sampling makes it difficult to distinguish between trunk and branch mutations in clinical specimens. Third, expression data is just as important as mutation data regarding multistep carcinogenesis, necessitating the use of multi-omics approaches, including WES and WGS sequencing, in clinical genome-based treatment. Overcoming these limitations will allow for the realization of a much more qualified anti-HCC strategy based on the genome profiling of clinical specimens.

## Figures and Tables

**Table 1:** Summary of treatment outcomes of recent treatment strategies.

	**Treatment**	**Publications**	**Study Name**	Objectives	5-Year Survival Rate	5-Year Recurrence Free Rate	OS	PFS (*;TTP)	ORR mRESICT/RECIST
Curative	RFA	Siina et al., 2005 [14]	NA	Size < 3 cm and Tumor number < 3	74% (4-year)	NA	NA	NA	NA
		Ng et al., 2017 [13]	NA	Size < 3 cm and Tumor number < 3	66.4%	18.3%	NA	NA	NA
	Hepatectomy	Ng et al., 2017 [13]	NA	Size < 3 cm and Tumor number < 3	66.5%	28.7%	NA	NA	NA
		Zhou et al., 2001 [15]	NA	Small HCC Large HCC	62.7%/37.1%	NA	NA	NA	NA
	Liver transplantation	Mazzaferro, et al. [12]	NA	Within Milan criteria	92% (4-year)	85% (4-year)	NA	NA	NA
Non-curative	TACE	Lencioni et al., 2016 [11]	SPACE	Intermediate	NA	NA	NR	5.5 *	NA
		Kudo et al., 2014 [10]	BRISK-TA	Intermediate	NA	NA	26.1	4.9 *	42%
	Sorafenib	Llovet et al., 2008 [17]	SHARP	Advanced: Unresectable HCC (1st line)	NA	NA	10.7–13.4	3.7–4.3	NA/2%
		Kudo et al., 2018 [20]	REFLECT						
		Finn et al., 2020 [9]	IMbrave150						
	Lenvatinib	Kudo et al., 2018 [20]	REFLECT	Unresectable HCC (1st line)	NA	NA	13.6	7.4	24.1%/18.8%
	Regorafenib	Bruix et al., 2017 [8]	RESORCE	Unresectable HCC (2nd line)	NA	NA	10.6	3.1	11%/7%
	Cabozantinib	Abou-Alfa et al., 2018 [7]	CELESTIAL	Unresectable HCC (2nd line)	NA	NA	10.2	5.2	NA/4%
	Ramucirumab	Zhu et al., 2019 [16]	REACH-2	Unresectable HCC (2nd line)	NA	NA	8.5	2.8	NA/5%
	Atezolizumab plus bevacizumab	Finn et al., 2020 [9]	IMbrave150	Unresectable HCC (1st line)	NA	NA	19.2	6.8	35.4%/29.8%

Representative outcome of major treatment strategies for HCC are summarized. Abbreviations: RFA: radiofrequency ablation; OS: overall survival; PFS: progression free survival; ORR: objective response rate; TTP: time to progression, the values with * in the TACE row means TTP

**Table 2:** Summary of the multistep accumulation of genomic and transcriptomic aberrations during hepatocarcinogenesis.

Author, Year	Methodology	Genetic Characteristics According to Each Phase through Multistep Hepatocarcinogenesis from Precancerous Liver Tissues to Advanced HCCs					
		Normal Liver	Cirrhosis	Dysplastic Nodule	Early HCC NIN-HCC Outer	Classical (Progressed) HCC NIN-HCC Inner	Advanced HCC
Brunner et al., 2019 [41]	WGS	CNV and SV: rare	*TERT*p mut not detected Mut in *ACVR2A, ARID2, ARID1A, TSC2, ALB,* etc. CNV and SV can be detected chromothripsis (3/9)	-	-	-	-
Zhu et al., 2019 [42]	WES, target seq	(F0 samples) low mutational burden HCC-related mut: not detected	*PKD1, KMT2D* *PPARGC1B, ARID1A* mut. liver protective mutation	-	-	-	-
Kim et al., 2019 [43]	WES, target seq	-	*TERT*p mut not detected (0/205) *ARID1A*(7/205), *ARID2*(4/205) *TP53*(6/205), *ATM*(4/205) etc. no CNV detected	-	-	*TERT*p mut (80%, 8/10)	-
Midorikawa et al., 2009 [44]	CGH array	-	1q gain, 1p LOH and 18p LOH: not detected	-	(NIN-HCC outer and eHCC) Gain on 1q (12/19) LOH on 1p and 18p (2/19)	(NIN-HCC inner and pHCC) Gain on 5q11.1-35.3 and 8q11.1-24.3 (14/25 and 16/25) LOH on 4q11-34.3 and 8p11.21-23.3 (10/25 and 12/25)	-
Midorikawa et al., 2020 [45]	WES, RNAseq, Methylation array	-	-	-	(eHCCs, N = 52) *TERT*p mut (65.3%) *TERT* overexpression (84.6%) *TP53/RB*1 mut (34.6%) *WNT* mut (23.0%)	(overt or progressed HCC, *n* = 108) *TERT*p mut (58.3%) *TERT* overexpression (74.0%) *TP53/RB1* mut (42.5%) *WNT* mut (43.5%) *CDH* inactivation, *EZH2* upregulation, 4q loss, 16q loss etc. (categorized into 4 groups according to the methylation status)	-
Takeda et al., 2020 [46]	WGS, target seq, CNV array	-	-	-	(NIN-HCC outer) *TERT* overexpression (8/8) due to HBV integration/gain, translocation, methylation and promoter mutation SV, chromothripsis detected	(NIN-HCC inner) *TERT* overexpression (4/5) due to the genetic aberrations as same as eHCC Wnt, MAPK, *and* mTOR associated gene mutations (case specific)	-
Nault et al., 2014 [47]	Sanger seq.	-	*TERT*p mut; not detected (0%, 0/172)	*TERT*p mut (6–19%, 5/48) no other mutations	*TERT*p mut (61%, 14/23) no other mutations	*TERT*p mut (42%, 7/17) other HCC-related mut (*CTNNB1, TP53*) (28%, 2/7)	*TERT*p mut (64%, 60/94) other HCC-related mut (*CTNNB1, TP53*) (56%, 20/35)

Major genetic aberrations in each phase of multistep hepatocarcinogenesis are summarized. Numbers within brackets after the description of each genetic alteration indicate the frequencies and/or numbers of samples with the corresponding genetic alteration among the total samples analyzed in the study. Abbreviations: CNV, copy number variation; mut, mutation; NIN, nodule-in-nodule; seq, sequencing; SV: structural variation; *TERT*p: *TERT* promoter; WES: whole-exome sequencing; WGS: whole-genome sequencing; NIN-HCC outer means the outer tumor of NIN-HCC, which is usually hypovascular well-differentiated HCC, while NIN-HCC inner means the inner tumor of NIN-HCC, which is hypervascular moderately or poorly differentiated HCC. NIN-HCC inner is considered more aggressive than NIN-HCC outer.
